# Biologic behavior and long-term outcomes of breast ductal carcinoma *in situ* with microinvasion

**DOI:** 10.18632/oncotarget.11639

**Published:** 2016-08-26

**Authors:** Yan Fang, Jiayi Wu, Wei Wang, Xiaochun Fei, Yu Zong, Xiaosong Chen, Ou Huang, Jianrong He, Weiguo Chen, Yafen Li, Kunwei Shen, Li Zhu

**Affiliations:** ^1^ Comprehensive Breast Health Center, Ruijin Hospital, Shanghai Jiaotong University School of Medicine, Shanghai 200025, P.R. China; ^2^ Department of Pathology, Ruijin Hospital, Shanghai Jiaotong University School of Medicine, Shanghai 200025, P.R. China

**Keywords:** breast carcinoma, ductal carcinoma in situ, microinvasion, clinico-pathological feature, prognosis

## Abstract

**Background:**

Ductal carcinoma *in situ* with microinvasion (DCIS-Mi) generally has favorable prognosis, but the long-term outcomes of DCIS-Mi and the biologic evolution from ductal carcinoma *in situ* (DCIS), DCIS-Mi, to DCIS with T1a breast cancer (DCIS-T1a) has not been specified. The aim of our study was to explore the biological and prognostic features of DCIS-Mi, compared with pure DCIS and DCIS-T1a.

**Results:**

After a median follow-up of 31 months, the 3-year estimated disease free survival(DFS) rate of DCIS-Mi patients was significantly lower than that of pure DCIS patients (89.5% vs 97.1%, P=0.009). Patients with DCIS-Mi or DCIS-T1a tumors had comparable 3-year estimated DFS rates (89.5% vs 94.3%, P=0.13). No significant difference in overall survival (OS) was found among different groups (99.6%, 100% and 99.1% for DCIS, DCIS-Mi and DCIS-T1a, P=0.797). In chemotherapy and trastuzumab-naive DCIS-Mi patients, human epidermal growth factor receptor2 (HER2) positivity (HR=21.8, 95%CI, 1.7-286.8, P=0.019) were independent predictor of worse DFS on multivariate analysis.

**Methods:**

During September 2002 and December 2014, 602 breast cancer patients who underwent radical surgery were retrospectively reviewed. Three hundred and fifty-nine patients (59.6%) had pure DCIS, 84(14.0%) and 159(26.4%) were diagnosed as DCIS-Mi and DCIS-T1a. Clinico-pathological features were compared between different subgroups.

**Conclusions:**

DCIS-Mi displayed a comparable survival to that of DCIS-T1a and a more aggressive biological nature than pure DCIS. Patients with HER2-positive DCIS-Mi had a worse survival and adjuvant chemotherapy plus target therapy needs to be further optimized in those patients.

## INTRODUCTION

According to the staging system of the American Joint Committee on Cancer (AJCC), ductal carcinoma *in situ* with microinvasion (DCIS-Mi) was defined as ductal carcinoma *in situ* (DCIS) with a microscopic focus of invasion ≤1 mm in the longest diameter [1], which is identified in 10–20% of DCIS cases and accounts for approximately 1% of all breast cancers [2, 3]. Due to the widespread application of screening mammography, the detection rate of both DCIS and DCIS-Mi significantly increased in recent years [4–6]. However, the natural history of cancer cells progression from DCIS to DCIS-Mi, and finally to invasive ductal carcinoma (IDC) remains unclear, and DCIS-Mi may represent the interim stage in the evolutionary progress from DCIS to IDC. There are no existing data focusing on comparison of DCIS, DCIS-Mi and DCIS with T1a tumors (DCIS-T1a) as a whole, and both overall survival and prognostic parameters of DCIS-Mi patients has yet not been specified. The current study aimed to compare the prognosis of patients with pure DCIS, DCIS-Mi and DCIS-T1a, as well as assess the prognostic clinico-pathological factors of DCIS-Mi.

## RESULTS

### Clinico-pathological features

The baseline clinico-pathological and treatment information differed by subgroups are shown in Table 1. The median age at initial diagnosis was 52 (range, 25–86) years old. Age and menopausal status distributed comparably in three groups. Tumor size seemed to have little impact on different stage of tumor progression and the incidence of axillary lymph nodes involvement significantly increased with the development of invasion (1.3%, 7.6%, 9.6% respectively, P<0.001). In pure DCIS, the proportion of estrogen receptor (ER)-positive tumors and progesterone receptor (PR)-positive tumors were 69.9% and 58.4% respectively, slightly higher than that of DCIS-Mi and DCIS-T1a. Of note, DCIS-Mi tumors tended to exhibit the highest proportion of human epidermal growth factor receptor2 (HER2)-positive tumor (29.5% for DCIS, 42.9% for DCIS-Mi and 29.3% for DCIS-T1a, P=0.048). In addition, Ki-67 index was higher in DCIS-Mi and DCIS-T1a than in pure DCIS.

**Table 1:** Baseline Characteristics of all Patients

Characteristics	Pure DCIS	DCIS-Mi	DCIS-T1a	P-value
	N=359(%)	N=84(%)	N=159(%)	
**Age(yrs)**				0.147
Age < 50	157(43.7)	27(32.1)	68(42.8)	
Age≥50	202(56.3)	57(67.9)	91(57.2)	
**Menopausal status**				0.156
Premenopausal	178(49.6)	32(38.1)	73(45.9)	
Postmenopausal	181(50.4)	52(61.9)	86(54.1)	
**Tumor size(cm)**				0.707
T≤2	207(61.8)	44(54.3)	85(57.4)	
2 < T≤5	116(34.6)	34(42)	58(39.2)	
T > 5	12(3.6)	3(3.7)	5(3.4)	
Unknown	24	3	11	
**Invasive foci**				
1		51(60.7)		
≥2		33(39.3)		
**Breast surgery**				0.042*
Mastectomy	273(76)	59(70.2)	133(83.6)	
Breast-conserving	86(24)	25(29.8)	26(16.4)	
**Lymph node status**				< 0.001*
Positive	4(1.3)	6(7.6)	15(9.6)	
Negative	293(98.7)	73(92.4)	141(90.4)	
Unknown	62	5	3	
**ER status**				< 0.001*
Positive	249(69.9)	42(50)	85(53.8)	
Negative	107(30.1)	42(50)	73(46.2)	
Unknown	3		1	
**PR status**				< 0.001*
Positive	208(58.4)	32(38.1)	66(41.8)	
Negative	148(41.6)	52(61.9)	92(58.2)	
Unknown	3		1	
**HER2 status**				0.048*
Positive	104(29.5)	36(42.9)	46(29.3)	
Negative	249(70.5)	48(57.1)	111(70.7)	
Unknown	6		2	
**Ki-67 Index(mean±SD)(%)**	14.29±14.26	18.25±14.65	19.12±15.89	0.002*
**Molecular subtypes**				< 0.001*
Luminal A	130(38.8)	19(22.6)	32(20.9)	
Luminal B	101(30.1)	23(27.4)	53(34.6)	
HER2 positive	72(21.5)	27(32.1)	35(22.9)	
Triple negative	32(9.6)	15(17.9)	33(21.6)	
Unknown	24		6	
**Chemotherapy**				< 0.001*
Yes	15(4.2)	16(19)	73(45.9)	
No	339(95.8)	68(81)	86(54.1)	
Unknown	5			
**Radiotherapy**				< 0.001*
Yes	55(15.4)	29(34.5)	32(20.1)	
No	302(84.6)	55(65.5)	127(79.9)	
Unknown	2			
**Endocrine therapy**				< 0.001*
Yes	138(39)	41(48.8)	92(57.9)	
No	216(61)	43(51.2)	67(42.1)	
Unknown	5			
**Targeted therapy (Trastuzumab)**				< 0.001*
Yes	/	3(3.6)	23(14.5)	
No	359(100)	81(96.4)	136(85.5)	

Abbreviation: Pure DCIS=Pure ductal carcinoma *in situ*. DCIS-Mi=Ductal carcinoma *in situ* with microinvasion. DCIS-T1a= Ductal carcinoma *in situ* with T1a invasive breast cancer. ER=estrogen receptor; PR=progesterone receptor; HER2=human epidermal growth factor receptor 2; SD=standard deviation.

* statistically significant.

As for IHC-based molecular subtype, pure DCIS were more likely to be luminal-like subtype (luminal A accounted for 38.8%, luminal B for 30.1%). With the procedure of invasion, the proportion of luminal-like subtype was decreasing (50% for DCIS-Mi and 55.3% for DCIS-T1a), whereas basal-like subtype was increasing (9.6% for DCIS, 17.9% for DCIS-Mi and 21.7% for DCIS-T1a). HER2-positive subtype in DCIS-Mi accounted for less of the overall population (32.1%) when compared with HER2-positive tumors (42.9%), mainly because HER2-positive tumors consisted of both luminal-B and HER2-positive subtypes.

The concordance of receptor status of DCIS-Mi between intraductal component and invasive component were also studied in 30 patients with adequate specimen for IHC test. Three patients (10%) with hormone receptor (HR)-positive *in situ* component displayed HR-negative invasive component, whereas none of the patients with HR-negative *in situ* component reversed to HR-positive in the invasive component. Two cases (6.7%) with HER2-positive DCIS showed HER2-negative microinvasive tumors and no cases experienced a negative-to-positve switch of HER2 status from intraductal to invasive component.

### Surgical management and adjuvant therapy

Patients with DCIS-T1a tumors were more likely to receive mastectomy than breast conserving surgery than those with pure DCIS or DCIS-Mi tumors (76.0% for pure DCIS, 70.6% for DCIS-Mi and 83.2% for DCIS-T1a).

When it came to adjuvant treatment, the proportion of use of chemotherapy increased from 4.2% in pure DCIS to 19.3% in DCIS-Mi and to 45.9% in DCIS-T1a (P<0.001), mainly in patients with TNBC, HER2-positive or node-positive tumors. Post-operative radiation therapy was given in 15.4%, 34.5% and 20.1% of the patients with pure DCIS, DCIS-Mi and DCIS-T1a. Most of the patients with HR-positive tumor in DCIS-Mi and DCIS-T1a groups received endocrine treatment, whereas the proportion in pure DCIS was much lower (39.0% for DCIS, 48.8% for DCSI-Mi and 57.9% for DCIS-T1a, respectively). Three patients (3.6%) among DCIS-Mi group received trastuzumab, which was administered in 23 patients (14.5%) in DCIS-T1a group.

### Survival information in overall population

**Figure 1:**
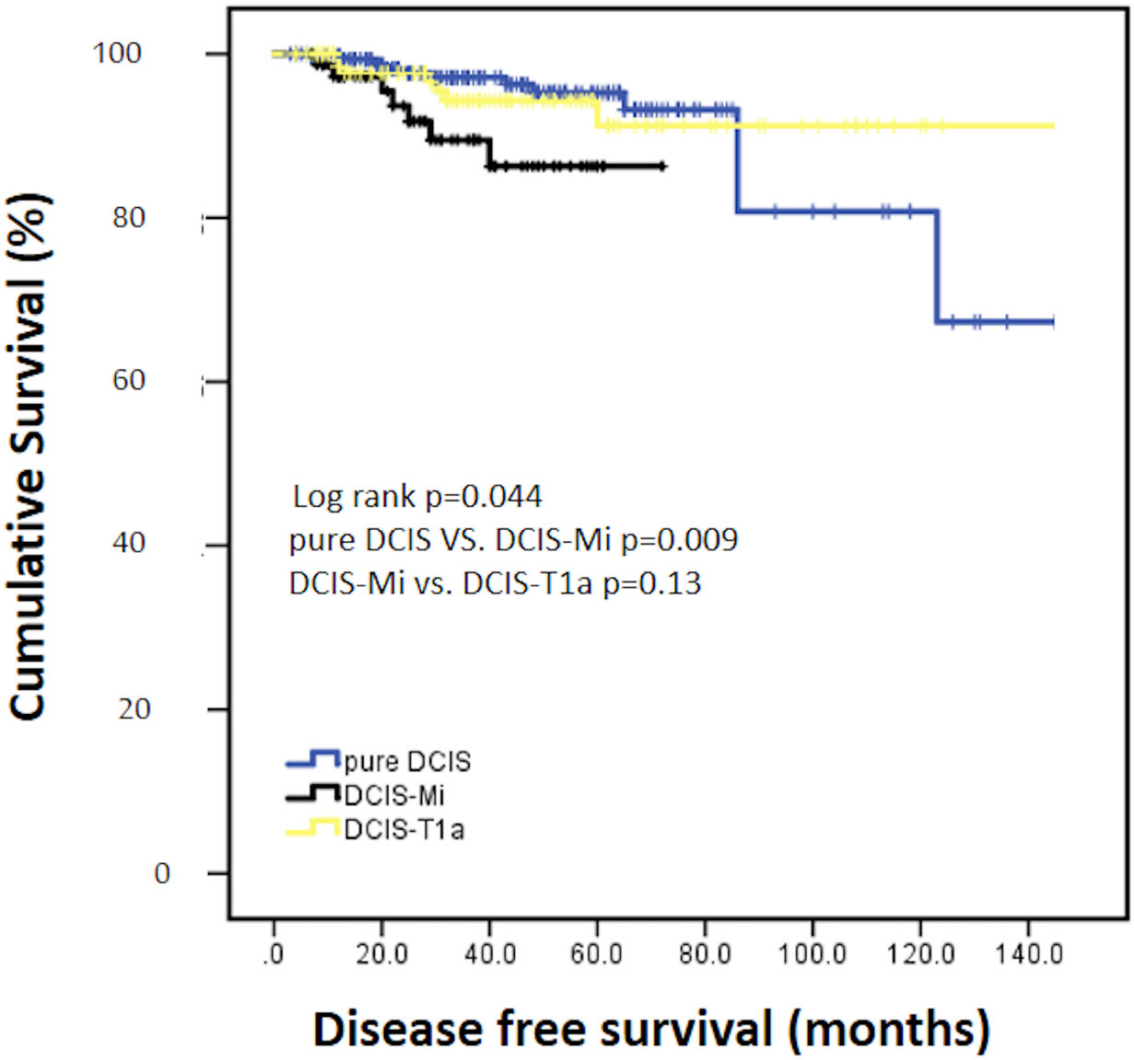
Disease free survival of different subgroups Pure DCIS=Pure ductal carcinoma *in situ*. DCIS-Mi=Ductal carcinoma *in situ* with microinvasion. DCIS-T1a=Ductal carcinoma *in situ* with T1a breast cancer.

**Figure 2:**
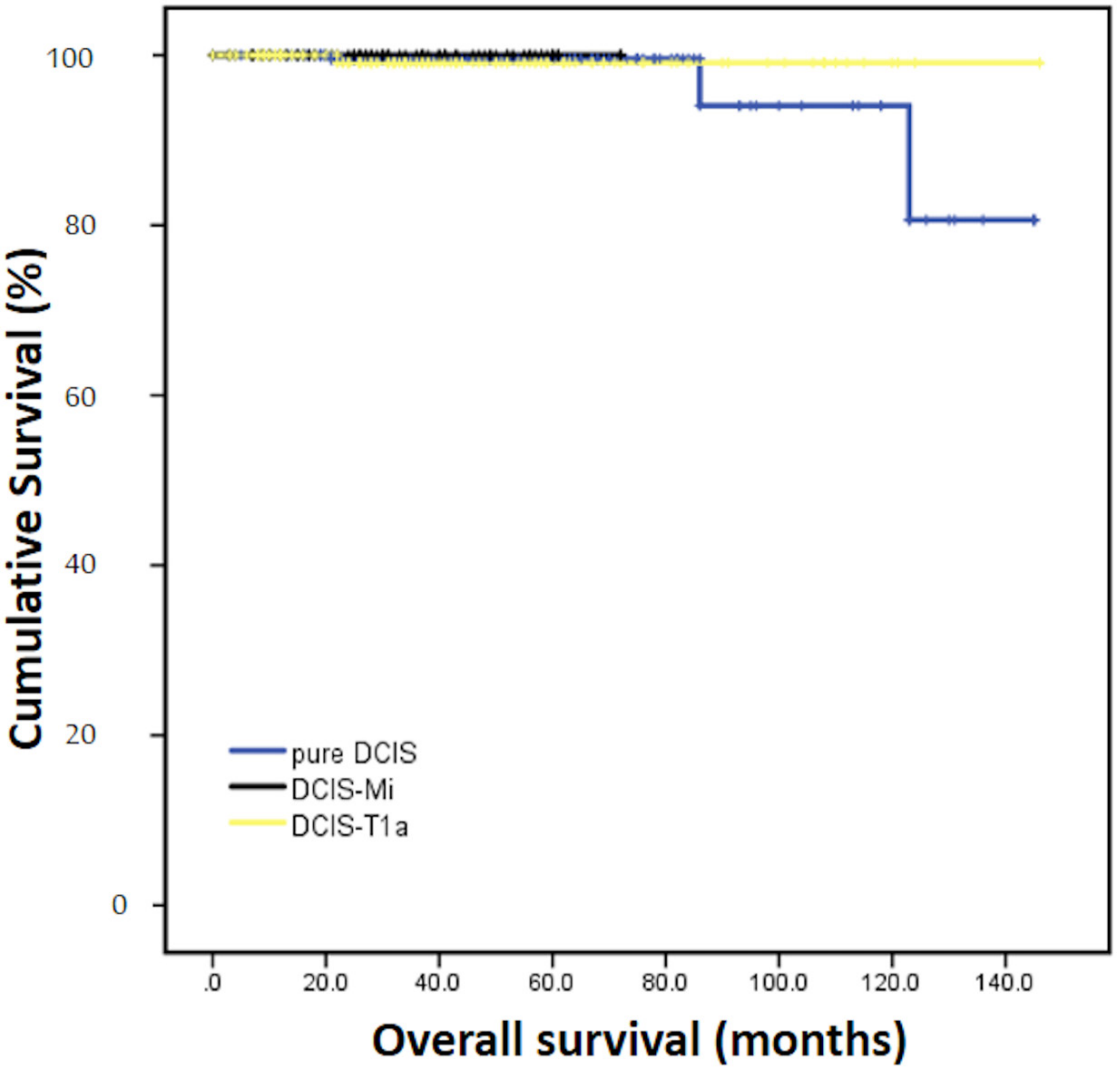
Overall survival of different subgroups Pure DCIS=Pure ductal carcinoma *in situ*. DCIS-Mi=Ductal carcinoma *in situ* with microinvasion. DCIS-T1a=Ductal carcinoma *in situ* with T1a breast cancer.

After a median follow-up of 31 months (range, 2-144), there were 28 DFS events totally. The 3-year DFS rates of pure DCIS, DCIS-Mi, DCIS-T1a were 97.1%, 89.5% and 94.3% (P=0.044). Patients with DCIS-Mi had significantly worse DFS when compared with DCIS patients (P=0.009), and no statistical significant difference was found between DCIS-Mi and DCIS-T1a groups (P=0.13, Figure 1). As for OS, no event was found in DCIS-Mi patients and the survival rates were comparable among three groups (99.6% for DCIS, 100% for DCIS-Mi and 99.1% for DCIS-T1a, P=0.797, Figure 2).

### Univariate and multivariate analyses of prognostic variables in DCIS-Mi patients

To further determine the independent prognostic factors of DFS in DCIS-Mi patients, both univariate and multivariate analysis were conducted.

In univariate model, young age was significantly associated with poorer DFS (P=0.019). Age, number of invasive foci, lymph node status, ER, PR and HER2 status were included in the multivariate analysis, and young age was the only independent prognostic factor for worse DFS (P=0.041, HR=5.747, 95%CI, 1.076-30.7, Table 2).

**Table 2:** Univariate and multivariate prognostic analysis of DFS for all DCIS-Mi patients

Characteristics	Univariate		Multivariate	
	Mean survival(months)	P value	HR(95%CI)	P value
**Age(yrs)**		0.019*		0.041*
< 50	49.5		5.8	
≥50	69.4		(1.1-30.7)	
**Invasive foci**		0.053		0.566
1	68.0		1.8	
≥2	51.0		(0.3-12.1)	
**ER status**		0.573		0.837
Positive	66.8		1.3	
Negative	55.2		(0.1-13.2)	
**PR status**		0.499		0.549
Positive	67.4		0.6	
Negative	55.2		(0.1-3.8)	
**HER2 status**		0.08		0.070
Positive	52.3		4.7	
Negative	68.8		(0.9-27.7)	
**Lymph node status**		0.519		0.548
Positive	55.2		0.5	
Negative	55.8		(0.04-5.4)	

Abbreviation: DFS=disease free survival. DCIS-Mi=Ductal carcinoma *in situ* with microinvasion. HR=hazard ratio. ER=estrogen receptor. PR=progesterone receptor. HER2=human epidermal growth factor receptor 2.

* statistically significant.

**Figure 3:**
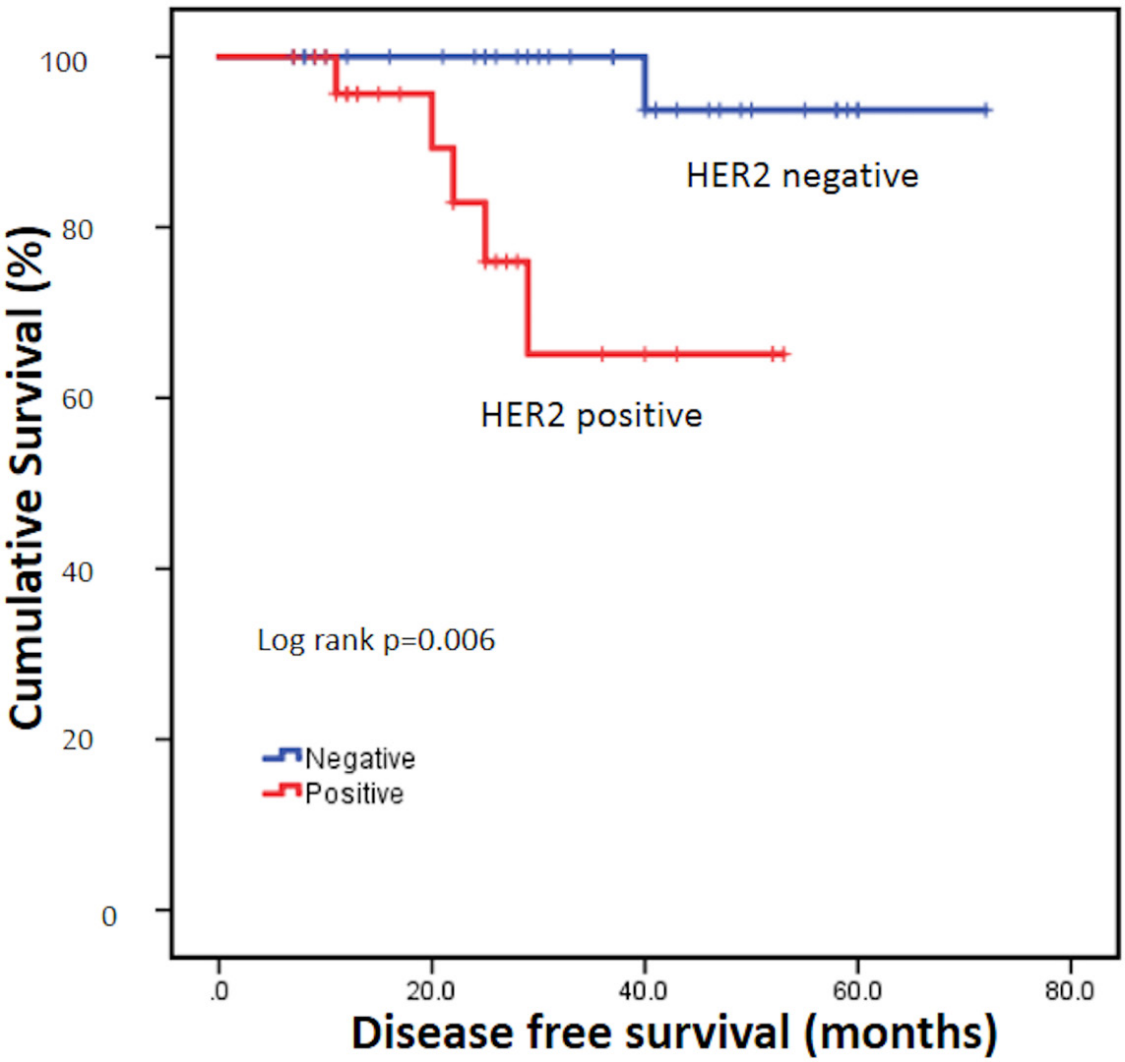
Disease free survival of different HER2 status HER2=human epidermal growth factor receptor 2.

To further clarify the natural history of DCIS-Mi, we excluded 16 patients treated with chemotherapy and trastuzumab and re-conducted univariate and multivariate analysis. In the remaining 68 patients, young age, multifocality and HER2 positivity were significantly associated with poorer DFS. In multivariate regression model, young age (P=0.021, HR=21.1, 95%CI, 1.6-281.6) and positive HER2 status (P=0.019, HR=21.8, 95%CI, 1.7-286.8) were independent prognostic factor for worse DFS (Table 3, Figure 3).

**Table 3:** Univariate and multivariate prognostic analysis of DFS for chemotherapy and trastuzumab-naive DCIS-Mi patients

Characteristics	Univariate		Multivariate	
	Mean survival(months)	P value	HR(95%CI)	P value
**Age(yrs)**		0.006*		0.021*
< 50	45.7		21.1(1.6-281.6)	
≥50	70.3			
**Invasive foci**		0.011*		0.754
1	67.4		1.52(0.11-20.80)	
≥2	33.0			
**ER status**		0.439		0.887
Positive	66.5		0.84(0.08-9.10)	
Negative	47.5			
**PR status**		0.433		0.123
Positive	67.0		0.16(0.02-1.65)	
Negative	47.7			
**HER2 status**		0.006*		0.019*
Positive	42.6		21.8(1.7-286.8)	
Negative	70.0			

Abbreviation: DFS=disease free survival. DCIS-Mi=Ductal carcinoma *in situ* with microinvasion. HR=hazard ratio. ER=estrogen receptor. PR=progesterone receptor. HER2=human epidermal growth factor receptor 2.

* statistically significant.

## DISCUSSION

Microinvasive carcinoma represents a less frequent subtype of breast cancer. Early data are beginning to elucidate the biologic underpinnings of patients with DCIS-Mi. However, due to the paucity and the non-uniformity of the clinical outcome data, it's still uncertain to separate DCIS-Mi from *in situ* carcinomas on one hand and, from small invasive carcinomas on the other. Previous studies have reported survival outcome of DCIS-Mi patients with conflicted results. Some indicated that DCIS and DCIS-Mi had similar survival, while others did not [3, 7]. With a relatively larger sample size, our study further indicated that DCIS-Mi exhibited a similar survival probabilities compared with DCIS-T1a and a more aggressive biology than DCIS.

Approximately 50~75% of DCIS were ER and/or PR-positive tumors, and reported expression rates of ER and/or PR in microinvasive carcinoma ranged from 50~68% [7–10], similar to the findings in our study. Expression of HR often correlated with low proliferation and better survival. In our study, the proportion of luminal like breast cancer decreased from pure DCIS, DCIS-Mi, to DCIS-T1a. Meanwhile, triple-negative subtype was more prevalent in DCIS-Mi and DCIS-T1a, whereas HER2-positive tumors were predominantly more frequent in DCIS-Mi than both DCIS and DCIS-T1a. Triple-negative and HER2-positive tumors are both known to be aggressive phenotypes, and their underlying and differing roles in cancer progression need more advanced research.

As for DCIS-Mi, according to AJCC staging system, it's suggested that pathologists should attempt to quantify the number of foci and the range of their sizes. We found that multifocality is associated with a worse survival in chemotherapy and trastuzumab-naive patients, though statistically insignificant in multivariate regression model. As we know, microinvasive carcinoma is nearly always encountered in a setting of DCIS (or, less often, lobular carcinoma *in situ*) where small foci of tumor cells have invaded through the basement membrane into the surrounding stroma. We postulated that the presence of multifocality of DCIS-Mi might be indicative of a driving force of DCIS to penetrate the basement membrane. Of note, rates of lymph node involvement considerably differed in DCIS-Mi with different number of foci (7.6% totally; 2.1% for one focus and 15.6% for multiple foci, P=0.037). Metastasis to axillary lymph nodes may represent the ability of invasion of cancer and contributed to the shortened DFS. Similar rate of lymph node metastasis was reported in a meta-analysis concerning sentinel lymph node biopsy in patients with DCIS-Mi [11]. We believe that this uncommon but unnegligible probability of lymph node metastasis warrant sentinel node biopsy in DCIS-Mi patients, especially in those with multiple microinvasions.

Up to now, there is no standard recommendation for the use of chemotherapy and target therapy in DCIS-Mi patients. We conducted survival analysis in patients free from adjuvant chemotherapy and trastuzumab, and found that HER2 status is an independent predictor for DFS. Previous studies had reported that HER2 overexpression was consistently correlated with poor survival in small, node-negative breast cancer [12–14]. There was an increase in the use of chemotherapy and trastuzumab among patients with HER2-positive T1a tumors over the past decade, especially after the report of the pivotal trial of trastuzumab in patients with small, node-negative, HER2-positive breast cancer [15, 16]. Our study indicated that even though the prognosis of DCIS-Mi is generally thought to be favorable, the HER2-positive subtype may still had aggressive biological behavior when compared to other subtypes. Optimization of adjuvant chemotherapy and target therapy in patients with HER2-positive DCIS-Mi tumors seems to be reasonable.

In conclusion, our study indicated that DCIS-Mi displayed a comparable survival to that of DCIS-T1a and a more aggressive biological nature than pure-DCIS. HR and HER2 status assessment on the microinvasive component, as well as sentinel node procedure, are justified in DCIS-Mi patients. Patients with HER2-positive DCIS-Mi had a worse survival and adjuvant chemotherapy and target therapy of DCIS-Mi needs to be further optimized.

## PATIENTS AND METHODS

### Patients

Six hundred and two female breast cancer patients who underwent radical surgery between September 2002 and December 2014 in Shanghai Ruijin Hospital were retrospectively reviewed. Three hundred and fifty-nine patients (59.6%) had pure DCIS, 84(14.0%) and 159(26.4%) were diagnosed as DCIS-Mi and DCIS-T1a. Each of them received breast-conserving surgery or total mastectomy with or without axillary node assessment (sentinel lymph node biopsy or axillary lymph node dissection) and finished adjuvant therapies after surgery according to physician's decision and/or the patient's preferences. No specific enrollment criteria of adjuvant treatment were required as for the primary endpoint of this study was not the efficacy of adjuvant therapy. Following data are also required: age at initial diagnosis, menopausal status, surgery type, pathologic tumor size and lymph node status, ER, PR, HER2 and Ki-67 index, adjuvant therapy and follow-up information.

### Pathological definition

Pure DCIS was classified as a neoplastic proliferation of epithelial cells confined to the mammary ductal-lobular system according to the World Health Organization Classification of Tumors. DCIS-Mi was defined as no invasive focus measuring >1 mm in a setting of DCIS according to the 7^th^ Edition of AJCC Cancer Staging Manual and DCIS-T1a as DCIS with invasive focus more than 1mm but not more than 5mm in greatest dimension. Immunohistochemical (IHC) features were assessed based on the invasive components in DCIS-Mi and DCIS-T1a groups while based on intraductal component in pure DCIS group. Status of ER, PR, HER2 and Ki-67 index was detected by IHC staining. ER and PR positivity was defined as no less than 1% positive tumor cells with nuclear staining. HER2 positivity was considered as HER2 3+ by IHC or positive on FISH, whereas cases with 0 to 1+ or 2+ without FISH positivity were regarded as negative. Ki-67 index was characterized as the proportion of positively nuclear staining cells among at least 1000 tumor cells in the area counted. Patients were subdivided into four different molecular phenotypes (luminal A-like, luminal B-like, triple negative and HER2-positive subtypes) using IHC markers according to 2013 St. Gallen Expert Consensus [18].

### Follow-up and statistic methods

Follow-up information and survival status was obtained through outpatient medical history of the patients and/or phone calls. DFS interval was defined as the time from the date of the diagnosis of breast cancer to the earliest occurrence of all local, regional, or distant recurrences and contralateral breast cancers, and any deaths. OS was defined as the time from the date of the diagnosis of breast cancer to any deaths whether they were breast cancer–related or not.

Pearson's Chi-square test (Fisher's exact test when necessary) was used to compare the distribution of clinico-pathological features between subgroups. DFS and OS were estimated using the Kaplan–Meier analysis, and the survival curves were compared using the log-rank test. Multivariate Cox regression analysis with stepwise selection was used to estimate the hazard ratio (HR), 95% confidence interval (CI), and the effects of the clinical and pathological variables. All statistical tests were two sided and P<0.05 was considered significant. The software package SPSS 22.0 was used for analysis.
